# Blood purification in sepsis and COVID-19: what´s new in cytokine and endotoxin hemoadsorption

**DOI:** 10.1186/s44158-022-00043-w

**Published:** 2022-04-04

**Authors:** Juan Carlos Ruiz-Rodríguez, Erika P. Plata-Menchaca, Luis Chiscano-Camón, Adolf Ruiz-Sanmartin, Ricard Ferrer

**Affiliations:** 1 Intensive Care Department, Vall d’Hebron University Hospital, Vall d’Hebron Barcelona Hospital Campus, Passeig de la Vall d’Hebron, 119-129 Barcelona, Spain; 2 Shock, Organ Dysfunction and Resuscitation Research Group, Vall d’Hebron Research Institute (VHIR), Vall d’Hebron University Hospital, Vall d’Hebron Barcelona Hospital Campus, Barcelona, Spain; 3 Departament de Medicina, Universitat Autonoma de Barcelona, Bellatera, Spain

**Keywords:** SARS-CoV-2 pneumonia, Sepsis, Cytokine hemoadsorption, Endotoxin Hemoperfusion, Polymyxin B, Cytosorb, Acute respiratory distress syndrome, Hypercytokinemia, COVID-19

## Abstract

Sepsis and COVID-19 are two clinical conditions that can lead to a dysregulated inflammatory state causing multiorgan dysfunction, hypercytokinemia, and a high risk of death. Specific subgroups of critically ill patients with particular characteristics could benefit from rescue treatment with hemoadsorption. There is a lack of adequately designed randomized controlled trials evaluating the potential benefits of cytokine or endotoxin hemoadsorption. Critically ill COVID-19 patients with severe acute respiratory failure poorly responsive to conventional treatment could be candidates to receive cytokine hemoadsorption in the presence of high levels of interleukin 6. This treatment can also be suitable for patients with refractory septic shock and hypercytokinemia. In the context of high endotoxin activity, hemoadsorption with polymyxin B could improve clinical parameters and the prognosis of patients with refractory septic shock. Predictive enrichment, using biomarkers or other individual features, identifies potential responders to cytokine, endotoxin, or sequential hemoadsorption. Besides, recognizing the particular subsets of patients likely to respond to one or both types of hemoadsorption will aid the design of future studies that accurately validate the effectiveness of these therapies.

## Background

Sepsis results from an inappropriate and dysregulated host response to infection, leading to an imbalance of pro-inflammatory and anti-inflammatory responses [1]. An excess of pro-inflammatory cytokines causes endothelial damage and systemic inflammatory response syndrome (SIRS). Severe cases progress to multiple organ failure and death [2]. Therefore, a rigorously regulated balance in the cytokine network is essential for controlling the infection and restricting excessive tissue-damaging inflammation. This network involves pro-inflammatory cytokines [tumor necrosis factor-alpha [TNF-α], interleukin 6 (IL-6), interleukin 1 (IL-1), interleukin 12 (IL-12), macrophage migration inhibitory factor (MIF), and interferon-gamma (IFN-γ)], anti-inflammatory cytokines [interleukin 10 (IL-10), interleukin 4 (IL-4), transforming growth factor-beta (TGF-β)], and soluble inhibitors of pro-inflammatory cytokines [3] [TNF receptor (TNFR), IL-1 receptor antagonist (IL-1Ra), and IL-2 receptor antagonist (IL-1R2)] [4, 5]. IL-10 and TGF-β decrease the production of pro-inflammatory mediators in immune cells and increase the production of IL-1Ra and sTNFRs [6, 7]. TNF-α and IL-1 are the primary mediators of inflammation-induced coagulation [8]. These two molecules amplify inflammatory cascades in an autocrine and paracrine manner by activating macrophages to secrete lipid mediators, other pro-inflammatory cytokines, and reactive oxygen and nitrogen species. These processes cause sepsis-induced organ dysfunction [1, 9]. TNF-α enhances the expression of adhesion molecules in the endothelium and increases the adhesiveness and extravasation of neutrophils into tissues [10, 11]. IL-6 induces fever [12] and is the central mediator of the acute phase of the inflammatory response [13, 14]. IL-6 binds to the soluble form of the IL-6 receptor. This complex combines with the signal-transducing component glycoprotein 130, which is present in various cells, including the endothelial cells, to elicit IL-6 signaling activation. However, IL-6 also has been shown to promote anti-inflammatory responses by inhibiting the release of TNF-α and IL-1 [15] and enhancing plasma levels of anti-inflammatory mediators [16–18].

### Cytokine hemoadsorption in sepsis

Several studies have found a relationship between IL-6 hypercytokinemia and organ dysfunction in sepsis, the response to conventional treatment, and prognosis [19]. In contrast, non-decreasing values or slowly progressive decreases of IL-6 levels have been observed in non-survivors [20, 21]. In a previous study [22], 82% of patients with community-acquired pneumonia and systemic elevation of cytokine levels, the patients with high levels of IL-6 and IL-10 were at increased risk for severe organ dysfunction [23, 24] and death [22, 24]. Other studies have reproduced these findings [20, 21, 23, 24]. IL-10 overproduction is also a strong predictor of severity and mortality [25, 26].

Given the central role of abnormal systemic inflammation in the pathophysiology of sepsis-induced organ dysfunction, the development of therapies modulating the cytokine storm could help improve immune homeostasis. Extracorporeal blood purification therapies have emerged as strategies to improve the outcome of sepsis patients, diminishing the systemic expression of pro- and anti-inflammatory mediators and immune homeostasis recovering [22]. These include various cytokine hemoadsorption techniques: (a) Cytosorb® (CytoSorbents Corporation, Monmouth Junction, NJ, USA); (b) oXiris® (Baxter, Meyzieu, France); (c) Alteco® LPS Adsorber (Alteco Medical AB, Lund, Sweden); (d) HA-330® and HA-380® (Jafron Biomedical Co., Zhuhai, Guangdong, China).

Cytosorb® is a highly bio- and hemocompatible cytokine adsorber approved for its use in clinical infectious and non-infectious conditions associated with increased levels of cytokines. The cartridge is the most used worldwide [27, 28]. The device comprises porous polymer beads within a vast and efficient surface area. It allows for adsorption and permanent binding of molecules in the 5–60 kDa range. This range includes most cytokines and other inflammatory molecules [29]. The primary mechanism involved in CytoSorb® cytokine adsorption is the passage of blood through polymer beads within a perfused cartridge by extracorporeal circulation [23]. This process can attenuate both the inflammatory response and aid recovery of balance much earlier. Its benefits have been addressed in different contexts and clinical conditions, such as sepsis, COVID-19, SIRS secondary to cardiopulmonary bypass, liver failure, and rhabdomyolysis-induced myoglobinemia [20, 21, 24].

There is a high burden of evidence from observational studies on the potential clinical benefits of using Cytosorb® in septic shock to reduce vasopressor support and mortality, though a high quality of evidence derived from randomized controlled trials evaluating the clinical benefits of cytokine depuration is scarce. Friesecke et al. [30] studied 20 consecutive patients with refractory septic shock and hypercytokinemia after 6 h of conventional treatment. Refractory septic shock was defined as a progressive shock despite full-conventional therapy, lactate levels ≥ 2.9 mmol/L (or increasing values compared to baseline), and high vasopressor requirements (noradrenaline dose > 0.3 mcg/kg/min). The mean IL-6 levels were 25.523 ng/mL (1052–491260). In this observational study, Cytosorb® therapy was associated with a significant decrease in vasopressor requirements and increased lactate clearance. This finding was associated with shock resolution in 13 patients. In another study of 45 patients with septic shock [31], a significant vasopressor dose reduction was observed in patients treated with cytokine hemoadsorption. Norepinephrine was reduced by 51.4%, epinephrine by 69.4%, and vasopressin by 13.9%. Also, a reduction in IL-6 levels by 52.3% and lactate levels by 39.4% was observed in the survivors. A survival rate of 75% was documented in patients who received treatment within 24 h of intensive care unit (ICU) admission. Sixty-eight percent of patients who received treatment within 24–48 h after ICU admission survived. In a retrospective study [32], Cytosorb® was associated with lower all-cause mortality at 28 days in patients with septic shock. Hawchar et al. [33] performed a proof of concept, prospective, randomized pilot study to assess the usefulness of Cytosorb® in 20 patients with early-onset septic shock. Cytosorb® reduced the need for vasopressor support. In a case-control study of septic shock patients [34] who received cytokine hemoadsorption with CytoSorb®, the median catecholamine requirements approximately halved within 24 h after the initiation of therapy. In-hospital mortality was significantly lower in the CytoSorb® group (35.7% *vs* 61.9%; *p* = 0.015). The benefits of cytokine hemoadsorption have been documented in the subgroup of patients who have refractory septic shock and very high levels of plasma cytokines (IL-6). Further studies are needed to determine the influence of hemadsorption in eliminating other substances. It can increase antimicrobial clearance, which could be clinically significant in the case of linezolid, teicoplanin, fluconazole, liposomal amphotericin B, and posaconazole [35].

**Fig. 1:**
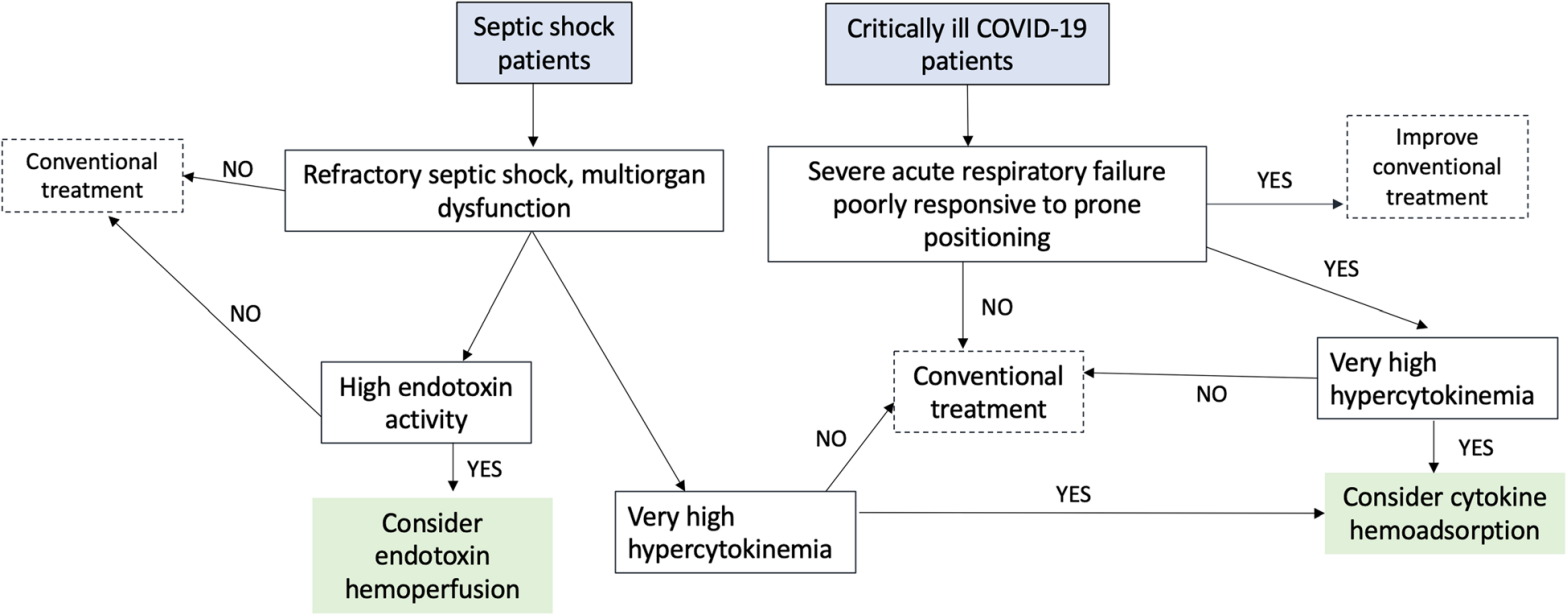
Potential applications of cytokine and endotoxin hemoadsorption in sepsis and COVID-19. *ECMO*: extracorporeal membrane oxygenation

Cytokine hemoadsorption may be beneficial as rescue therapy in the subgroup of patients with refractory septic shock, hyperlactatemia, multiorgan failure, and very high hypercytokinemia (Fig. 1). This strategy does not necessarily imply acting when multi-organ dysfunction is already established, as there is a risk of losing a valuable therapeutic window to change outcomes. The optimal timing to start cytokine hemoadsorption is not well defined. Earlier actions, particularly before the development of overt renal failure, have been shown to be safe and have significant effects in reducing norepinephrine requirements. Predictive enrichment in well-designed randomized controlled trials should be performed to validate the usefulness of cytokine hemoadsorption.

### Cytokine hemoadsorption in COVID-19

The extracorporeal cytokine hemoadsorption cartridge, Cytosorb®, was approved for its use in critically ill COVID-19 patients [36, 37]. The device has been previously used for cytokine storm-related hyperinflammatory conditions [38] and has been subject to many recent studies [39]. In the medical literature, cytokine storm refers to a heterogeneous group of disorders characterized by life-threatening hyperinflammation [40].

Since COVID-19 presents three distinct stages of disease progression, there are several challenges when managing patients [41]. The different clinical profiles correspond to different clinical stages, individual responses to therapy, and prognoses [42]. The following three stages determine the severity of COVID-19: early, pulmonary, and hyperinflammatory. The hyperinflammatory phase of COVID-19 is characterized by a multisystemic inflammatory syndrome, high levels of inflammatory biomarkers (the so-called “cytokine storm”), and an increased risk of organ dysfunction and death [43, 44]. Since early reports from China, the cytokine storm was recognized as the primary clinical feature associated with the severity of the disease [45].

There is accumulating evidence that the resulting inflammatory response in COVID-19 is not homogeneous throughout the disease [46, 47]. During the initial asymptomatic phase, hypercytokinemia is not clinically evident. In later stages, during the hyperinflammatory state, the massive cytokine release, which begins within the first 24–48 h of disease onset, causes a worsening of disease progression and severity that becomes evident after 7 to 10 days of infection [48]. During this stage, clinical deterioration is ubiquitous, and overt acute respiratory failure appears, as dysregulated immune response leads to diffuse alveolar damage, hyaline membrane formation, thrombus formation, fibrin exudates, and fibrotic healing [49]. These mechanisms lead to acute respiratory distress syndrome (ARDS) [50], whose frequency is up to 26% in SARS-CoV-2 infection [51, 52].

Potentially useful rescue therapies do not fit all COVID-19 patients. The indication for delivering cytokine hemoadsorption as adjuvant treatment in critically ill COVID-19 patients should be individualized [53]. Avoiding immunosuppression is the primary goal during the initial stages, as high viral loads are present. Thus, patients presenting a mild-moderate disease do not benefit from adjuvant therapies, such as cytokine hemoadsorption. In subsequent advanced stages, immunomodulation is the cornerstone for treatment [54, 55]. There are two distinct clinical phenotypes of COVID-19 patients [55]. The first phenotype is characterized by a mild-moderate disease and low viral loads, in which preserved interferon responses, regulated production of cytokines, and rapid recovery from initial lymphopenia are observed. Instead, a particular subset of patients presents the second phenotype, characterized by a severe disease with a high risk of death, high viral loads and cytokine plasma levels, impaired interferon response, and sustained lymphopenia. We indicate cytokine hemoadsorption in patients with severe acute respiratory failure refractory or poorly responsive to prone positioning in the context of a hyperinflammatory state (determined by very high levels of biomarkers, such as IL-6, ferritin, and D-dimer) (Fig. 1).

Several organizations support the use of cytokine hemoadsorption in patients with severe SARS-CoV-2 infection, even though the clinical experience is scarce and comes mainly from case reports and some case series [56–58]. The National Health Commission and National Administration of Traditional Chinese Medicine have recommended cytokine hemoadsorption with Cytosorb® as adjuvant treatment for severe and critical COVID-19 [59]. The Brescia Renal Covid Task Force supports the use of Cytosorb® hemoadsorption in COVID-19 patients with ARDS or acute kidney injury requiring continuous renal replacement therapy (CRRT) [60]. The Panamanian Association of Critical Medicine and Intensive Therapy recommends using Cytosorb® in patients with hyperlactatemia and high-dose vasopressors, not responding to conventional therapy. Also, patients with severe ARDS and high respiratory support requirements are considered [61]. The Colombian consensus recommends cytokine hemoadsorption in patients with cytokine storm and lack of response to treatment [62]. In April 2020, the Food and Drug Administration issued an Emergency Use Authorization for Cytosorb® to treat patients 18 years of age or older with confirmed COVID-19 admitted to the ICU, who have established or imminent respiratory failure, severe disease, or life-threatening disease (e.g., respiratory failure, septic shock or multiorgan dysfunction) [36].

Large-scale studies evaluating cytokine hemadsorption in critically ill COVID-19 patients are lacking, and some have introduced important selection bias when sampling from heterogeneous populations of critically ill patients. Rampino et al. [63] published a case series of 9 consecutive critically ill COVID-19 patients requiring continuous positive airway pressure. In their study, all patients who received the treatment survived, and only two needed endotracheal intubation. The inclusion criteria were confirmed SARS-CoV-2 pneumonia, in addition to a P_a_O_2_/F_i_O_2_ ratio < 200 mmHg, C-reactive protein levels > 10 mg/dL, and a lymphocyte count < 1500/mm^3^. In another study [64], the authors delivered hemoadsorption with Cytosorb® for 24 to 48-h-sessions to 11 critically ill COVID-19 patients who required invasive mechanical ventilation due to rapidly progressive ARDS. Nassiri et al. [65] used Cytosorb® in 26 critically ill COVID-19 patients with moderate ARDS (P_a_O_2_/F_i_O_2_ ratio < 200), C-reactive protein > 50 mg/l and ferritin > 1500 mcg/l). Overall, 46.2% of patients received mechanical ventilation. Paisey et al. [66] studied 15 patients with severe COVID-19 who received cytokine hemoadsorption (HA-330 cartridges were used in 5 patients, and Cytosorb® adsorbents in 10). All patients were on invasive mechanical ventilation and CRRT, and 11 received extracorporeal membrane oxygenation (ECMO) support. In a multicenter study [67], the authors evaluated the response of 37 mechanically ventilated patients who had received cytokine hemoadsorption using the oXiris® membrane. The indication for hemoadsorption was systemic inflammation associated with AKI, hemodynamic instability, or multiorgan dysfunction.

Another potential application of cytokine hemoadsorption is the adjunctive therapy in pediatric patients with multiorgan dysfunction due to multisystem inflammatory syndrome in children (MIS-C) associated with COVID-19. In a previous report [68], rapid improvements in shock and multiorgan dysfunction parameters were achieved, and cytokine levels (IL-6 and IL-10) decreased considerably. C-reactive protein, soluble CD25 [sCD25], and ferritin levels decreased following the attenuation of hyperinflammation. Although causality could not be confirmed because other treatment interventions could have contributed to a good outcome, a temporal relationship between the initiation of hemoadsorption and clinical improvement was evident. Rapid improvements in organ function were documented 24 h after initiation of therapy.

Clinicians should consider the undesirable effects of hemoadsorption. Alterations in the pharmacokinetics of some antibiotics, such as teicoplanin, have been described [69]. Therefore, it is necessary to monitor antibiotic concentrations, particularly when prolonged sessions of hemoadsorption are indicated. Regarding infectious complications, hemoadsorption sessions are of short duration, and the risk of infection of intravascular devices used for extracorporeal support is low, particularly in critical care units where preventive measures are widely implemented. Previous experience in sepsis showed that cytokine hemoadsorption is a safe procedure with no associated adverse effects [70]. Hemoadsorption-associated infections have not been described.

Critically ill COVID-19 patients with severe ARDS refractory to prone positioning and hypercytokinemia can receive adjuvant treatment with cytokine hemoadsorption [71]. Also, children with MIS-C and multiorgan dysfunction could be candidates for receiving this adjuvant treatment. Cytokine hemoadsorption therapy is a promising intervention for immunomodulation in patients with severe ARDS. Further studies and well-designed randomized controlled trials should be conducted to accurately set the indications and clinical benefits of cytokine hemoadsorption in COVID-19.

### Baseline biomarker levels and the clinical response to cytokine hemadsorption

Critically ill COVID-19 patients do not present increased plasma levels of biomarkers as other subsets of critically ill patients (e.g., septic shock or sepsis with ARDS patients). Previous studies have found mild to moderate elevations of C-reactive protein, IL-6, and ferritin [72]. However, there are no established thresholds of inflammatory biomarker levels to recommend cytokine hemoadsorption. Individual responses are heterogeneous, and numerous underlying factors affect biomarker levels. Thus, the real possibility to establish valid thresholds is uncertain. Damiani et al. [64] showed the median values of IL-6 to indicate hemoadsorption were 355 pg/mL (interquartile range, IQR, 263–466), 118 pg/mL (IQR 19–221, *p* = 0.003) at the end of therapy and 169 pg/mL (IQR 61–253, *p* = 0.03) after 24 h of therapy. The authors found a significant decrease in C-reactive protein and P_a_O2/FiO2 ratio improvements. Similar findings were reported in another study [65], in which P_a_O_2_/F_i_O_2_ ratio, sequential organ function assessment (SOFA) score, and inflammatory biomarkers (procalcitonin, CRP, and ferritin) improved significantly. The mortality rate was 19.2%. Cytokine hemoadsorption can reduce ferritin, C-reactive protein, procalcitonin, and lactate levels [66].

Nevertheless, no significant differences have been found in studies documenting mild to moderate increases in baseline IL-6 and IL-10 levels before treatment [66]. Villa et al. [67] initiated cytokine hemoadsorption after 3 to 4 days from ICU admission and 2 weeks of symptom onset. The reductions in IL-6 concentrations were significant during the first 24 of treatment. Baseline levels were high [1230 pg/ml (IQR 895) that diminished to 479 pg/ml (IQR 531) at 24 h of treatment initiation, 320 pg/ml (IQR 259) at 48 h, and 160 pg/ml (IQR 141) at 72 h (*p* = 0.001)]. Organ function and the risk of death improved following reductions in biomarker levels.

Some studies do not support the use of Cytosorb® in critically ill COVID-19 patients. The CYCOV trial [73] included 34 patients with severe COVID-19 pneumonia requiring ECMO. Seventeen patients received cytokine hemoadsorption for 72 h. There were no significant differences in IL-6 levels between the two groups after 72 h of treatment. This finding is explained by the low median baseline IL-6 concentrations that decreased from 357 pg/mL (IQR 177.4–118.0) to 98.6 pg/mL (71–192.8) in the cytokine adsorption group and from 289.0 pg/mL (87–787.0) to 110.0 pg/mL (48–198.5) in the control group. The results of the CYVOV trial are not comparable with the results of ongoing studies recruiting patients with highly increased IL-6 levels, as the rate of cytokine elimination by hemoadsorption depends on the presence of baseline high plasma levels of cytokines [22]. Moreover, the strategy for delivering cytokine hemadsorption also influences outcomes. Previous studies used fixed hemoadsorption regimens overlooking changes in IL-6 levels during therapy. Real-time IL-6 levels can be measured during the hemoadsorption sessions to withhold the treatment when IL-6 levels decrease, and the patient improves.

### Endotoxin hemoadsorption with polymyxin B in sepsis

Endotoxin is a lipopolysaccharide (LPS) is a component present in the outer membrane of gram-negative bacteria. Its presence results in the elevation of pro-inflammatory and anti-inflammatory cytokines [74], activating the innate immune response and mediating the clinical manifestations of early sepsis. LPS elicits its actions through the transmembrane receptor toll-like receptor 4, expressed on innate immune system cells. The LPS-binding protein (LBP) carries circulating endotoxin and facilitates its recognition by the cell through receptor CD14. Recognition of the LPS-LBP complex transduces the intracellular endotoxin signal to the cell nucleus, resulting in the expression of a vast and complex network of inflammatory mediators, activation of macrophages, neutrophils, endothelial cells, and the coagulation cascade [75, 76].

Endotoxin activity is a valuable biomarker of disease severity. The lipid-A domain of endotoxin is the primary cause of the toxicity associated with LPS. The clinical syndrome is characterized by fever, diarrhea, hemodynamic instability, multiple organ failure, and death [77]. There is a tight correlation between endotoxin levels and severity of septic shock, organ dysfunction, and the risk of death [76]. Up to 82% of patients with septic shock have endotoxemia, showing intermediate or high endotoxin activity [78]. High endotoxin activity is associated with significantly high lactate concentration levels and the need for high-dose inotropes.

However, the measurement of human endotoxin is troublesome. The first diagnostic test available was the chromogenic *Limulus* amebocyte lysate assay that provided indirect endotoxin activity measures [79]. However, the assay is not specific for endotoxin, as other microbial products, especially fungi, can activate the *Limulus* reaction. Subsequently, in 2004, the endotoxin activity assay (EAA) was developed, a chemiluminescent rapid (30 min) assay described by Romaschin in 1998 [80]. It is based on the ability of an antibody to assemble an antibody-antigen complex in whole blood. This antibody targets the lipid A epitope of endotoxin. It has very high sensitivity due to its high binding affinity. Outstandingly, the antibody does not cross-react with fungal or gram-positive components (has high specificity). The assay results are expressed in EAA units (< 0.39 is considered low, 0.40–0.59 intermediate, 0.60–0.89 high, and > 0.9 very high or extreme). This assay uses neutrophils as a readout system; therefore, storing specimens for later assaying is impossible. Measurements must be performed within 3 h of collecting the sample. The EAA is the only assay approved by the Food and Drug Administration of the USA for measuring endotoxin activity in whole blood.

Consequently, endotoxin has been considered a therapeutic target in critically ill patients with sepsis and septic shock. Hemoadsorption with a fiber column immobilized with polymyxin B (PMX) (Toraymyxin®; Toray, Tokyo, Japan) is one of the best-known endotoxin depuration strategies. Another endotoxin and cytokine absorptive device is the oXiris® hemofilter (Baxter, Meyzieu, France).

Various clinical trials have evaluated the clinical efficacy of endotoxin hemoadsorption in septic shock. In a pilot study, 36 surgical patients with severe sepsis or septic shock due to intraabdominal infection were randomized to receive endotoxin hemoadsorption with PMX over 2 h (*n* = 17) or conventional therapy (*n* = 19) [81]. There were no statistically significant differences in EAA from baseline to 6, 8, or 24 h of treatment initiation between the two groups. Five of the eighteen (28%) patients in the control group and five of the seventeen (29%) patients in the PMX group died during the study period. There were no statistically significant differences in survival, the mean duration of ICU stay, or the number of ICU-free days between the two groups. However, PMX hemoperfusion improved cardiac and oxygen delivery indices and decreased the need for CRRT.

The studies evaluating the clinical effects of endotoxin hemadsorption have shown that the treatment is well-tolerated with virtually no significant side effects. In the previously mentioned study, the use of a PMX cartridge was safe and could improve cardiac and renal dysfunction. The EUPHAS trial [82] evaluated hemodynamic improvements with PMX hemoperfusion in 64 patients with intraabdominal infection-related severe sepsis and septic shock. The treatment was effective in reducing the dose of vasopressors and SOFA scores. PMX hemoadsorption decreased the 28-day mortality rate in the intervention group (32%) compared to the standard treatment group (53%). In the ABDOMIX trial [83], the authors included 243 patients who developed septic shock within the 12-h postoperative period after emergency laparotomy for secondary peritonitis. The PMX hemoperfusion group (*n* = 119) received two sessions of hemoadsorption in addition to conventional treatment. There were no significant differences in the SOFA score and 28-day mortality rate between the intervention and control groups (27.7% vs. 19.5%). A total of 220 sessions were performed, and early interruption of the first treatment session was registered in 25 cases (11%) due to circuit clotting. A total of two PMX hemoperfusion sessions were delivered in only 81 of 119 patients (69.8%). None of the previously discussed studies performed plasma EAA levels.

The EUPHRATES trial [84] was a well-performed randomized controlled trial with a large sample of patients and sufficient scientific rigor. This study used EAA for predictive enrichment in the selection of patients. The authors included 450 critically ill patients with septic shock and an EAA level of 0.6 or higher. The intervention group received PMX hemoperfusion (90–120 min sessions) in addition to standard therapy. The sessions were completed within 24 h of study enrollment (*n* = 224). The control group received simulated or “sham” hemoperfusion plus standard treatment (*n* = 226). PMX hemoperfusion did not significantly reduce the 28-day mortality at the end of the study. However, a post hoc analysis of the EUPHRATES trial [85], including 194 patients with EAA between 0.6 and 0.89, demonstrated a survival benefit from PMX hemoperfusion. Monti et al. [86] published the first study evaluating PMX hemoperfusion as rescue therapy in 52 patients with refractory septic shock poorly responsive to conventional therapy. The median SOFA score was 10 (8–14), and the serum lactate level was 5.89 ± 4.04 mmol/L. All patients were mechanically ventilated, and 90% had received corticosteroids. Rapid improvement in organ dysfunction was achieved after treatment. The overall 30-day mortality was lower (29%) than estimated by the acute severity scores (47%).

Accordingly, the subset of critically ill patients with refractory septic shock, severe multiorgan dysfunction, adequate source control, and EAA 0.6–0.9 are suitable candidates for endotoxin hemoadsorption. A prospective, multicenter, randomized, open-label trial of standard medical care plus the PMX cartridge versus standard medical care alone, the TIGRIS study [87], is currently recruiting critically ill patients with septic shock and EAA within the range of ≥ 0.60 to < 0.90. Eligible and consented participants are randomized to receive either two sessions of PMX hemoperfusion (for 1½ to 2 h per treatment session approximately 24 h apart) plus standard medical care or standard medical care alone. Their mortality status is assessed at 28 days of treatment. Follow-up is done for up to 12 months after enrollment.

### Sequential hemoadsorption

Recent experience has shown that hemoadsorption aids the recovery of immune homeostasis. However, in some patients, endotoxin-only adsorption may be insufficient [88]. Endotoxemia and the overproduction of inflammatory mediators, in the form of cytokine storm, are paramount for the severity of sepsis and septic shock and determine prognosis [21, 89, 90]. Sequential hemoadsorption (endotoxin hemoadsorption with PMX, Toraymixin®, and subsequent cytokine hemoadsorption with Cytosorb®) has been applied in highly selected patients [91]. Precision medicine has allowed for a better selection of individuals according to their phenotypic and genetic profile to identify patients who could benefit from sequential hemadsorption (cytokine and endotoxin hemoadsorption). The candidates for sequential hemoadsorption are patients with refractory septic shock, multi-organ dysfunction, high endotoxemia, and hypercytokinemia (extremely high levels of IL-6). Real-time monitoring of plasma cytokines (IL-6, IL-10) can guide clinicians to withhold therapy [71]. The persistence of high levels of IL-10 is a valuable biomarker of a state of immunoparalysis [92]. Sequential hemoadsorption is intended to remove the primary stimulus that induces the dysregulated inflammatory response.

Hybrid therapies, such as the combined use of endotoxin hemoadsorption and coupled plasma filtration in a single circuit [93], have been studied in a particular group of cardiac surgery patients complicated with sepsis and EAA levels > 0.6. However, some researchers have excluded patients with high vasopressor requirements and acute severity scores in their studies. The presence of adequate drainage of infection source and the severity profile of patients should be considered before using hybrid hemoadsorption as adjunctive therapy.

We have clinical experience in sequential hemoadsorption with Toraymyxin® and Cytosorb, but other researchers have applied different approaches. Rossetti et al. [94] approach in pediatric patients proposes a double hemoadsorption using the CRRT circuit with the oXiris® membrane in association with two runs of Toraymyxin. Besides describing sequential techniques, novel advances have shown the scientific community that complementary hemoadsorption strategies are plausible to achieve homeostasis, are safe and have no adverse effects.

## Conclusions

Blood purification remains an alternative for rescue treatment in the most severe cases with features that make them suitable to improve after treatment. Critically ill COVID-19 patients with severe ARDS refractory to prone positioning and hypercytokinemia can be considered to receive adjuvant treatment with cytokine hemoadsorption. Endotoxin and cytokine hemoadsorption remain a suitable rescue therapy for subsets of sepsis patients with high endotoxemia or hypercytokinemia and multiorgan dysfunction. Further studies and well-designed randomized controlled trials should be conducted to accurately set the indications and clinical benefits of cytokine hemoadsorption in COVID-19. Predictive enrichment should be used to improve the future design of trials evaluating the role of blood purification in sepsis.

## Data Availability

Not applicable.

## Abbreviations

IL-6: Interleukin 6
IL-10: Interleukin 10
ICU: Intensive care unit
ARDS: Acute respiratory distress syndrome
CRRT: Continuous renal replacement therapy
MIS-C: Multisystem inflammatory syndrome in children
ECMO: Extracorporeal membrane oxygenation
SOFA: Sequential organ function assessment
EAA: Endotoxin activity assay
PMX: Polymyxin B
